# Wells criteria for DVT is a reliable clinical tool to assess the risk of deep venous thrombosis in trauma patients

**DOI:** 10.1186/s13017-016-0078-1

**Published:** 2016-06-08

**Authors:** Shrey Modi, Ryan Deisler, Karen Gozel, Patty Reicks, Eric Irwin, Melissa Brunsvold, Kaysie Banton, Greg J. Beilman

**Affiliations:** North Memorial Medical Center, Robbinsdale, MN USA; Department of Surgery, University of Minnesota Medical Center, Minneapolis, MN USA

**Keywords:** DVT risk assessment, Wells score, Trauma patients

## Abstract

**Background:**

Deep Vein Thrombosis (DVT) is a common complication in trauma patients. Venous duplex surveillance is used widely for the diagnosis of DVT, however, there is controversy concerning its appropriate use. The Wells criterion is a clinically validated scoring system in an outpatient setting, but its use in trauma patients has not been studied. This study evaluated the application of the Wells scoring system in trauma population.

**Methods:**

Wells scores were calculated retrospectively for all patients who were admitted to the trauma service and underwent Venous Duplex Scanning (VDS) at the author’s institution between 2012 and 2013. Correlation of Wells score with DVT and its efficacy in risk stratifying the patients after trauma was analyzed using linear correlation and receiver operating characteristic (ROC) curve. Sensitivity and specificity of Wells score in ruling out or ruling in DVT were calculated in various risk groups.

**Results:**

Of 298 patients evaluated, 18 (6 %) patients were positive for DVT. A linear correlation was present between Wells score and DVT with *R*^*2*^ = 0.88 (*p* = 0.0016). Median Wells score of patients without DVT was 1 (1–3) compared to a median score of 2 (1–5) in those with DVT (*p* < 0.0001). In low risk patients (scores <1), Wells scoring was able to rule out the possibility of DVT with a sensitivity of 100 % and NPV of 100 %, while in moderate-high risk patients (scores ≥2), it was able to predict DVT with a specificity of 90 %. Area under ROC curve was 0.859 (*p* < 0.0001) demonstrating the accuracy of Wells scoring system for DVT risk stratification in post trauma patients.

**Conclusions:**

A Wells score of <1 can reliably rule out the possibility of DVT in the trauma patients. Risk of developing DVT correlates linearly with Wells score, establishing it as a valid pretest tool for risk stratification.

**Electronic supplementary material:**

The online version of this article (doi:10.1186/s13017-016-0078-1) contains supplementary material, which is available to authorized users.

## Background

Venous thromboembolism (VTE), comprising of deep vein thrombosis (DVT) and pulmonary embolism (PE), is the third leading cause of death in hospitalized trauma patients, with an estimated incidence of 5–20 % with prophylaxis [1–3]. This wide range in incidence of VTE is attributed to variability in patients’ risk factors, choice of prophylaxis and modalities of screening and detection of VTE [1, 4]. Thromboprophylaxis in trauma patients is complex for many reasons, one of them being the presence of an early coagulopathy present in 25 % of trauma patients at the time of admission [5], which is further complicated by hypo-perfusion, acidosis and resuscitative measures [4, 6]. This coagulopathy shifts to a pro-thrombotic state early after traumatic injury necessitating thromboembolism prophylaxis. Additionally, these patients have a high bleeding risk associated with the use of anticoagulants and limitation of use of compression devices due to extremity injuries [7]. DVT has been chosen as an important indicator of quality of care and pay for performance criteria, with an underlying assumption that all DVTs are preventable with appropriate detection and prophylaxis [8]. This has led to an increased use of duplex ultrasound scanning for DVT in asymptomatic trauma patients, which may not be cost efficient or at times effective in preventing clinically relevant VTEs in a subset of these patients [7, 9]. Furthermore, there is immense variability in the utilization of duplex ultrasound screening among various trauma centers in the National Trauma Data Base (NTDB) [8]. Currently, screening is suggested for patients who are at high risk of developing DVT and have received suboptimal or no thromboprophylaxis. There are, however multiple opinions on what factors define a high risk trauma patient [1, 8] or what constitutes an optimal regimen for use in screening or prophylaxis against DVTs. Given this clinical dilemma, a means of increasing the pretest probability of screening algorithms is needed to optimize DVT detection and cost-effectiveness. Multiple tools are available to identify high-risk patients in the outpatient setting such as Wells Score, Geneva Score, Minaiti Score and Charlotte rule, of which the Wells score with its modification is the most widely used and accepted scoring system [10–12].

The aim of the current study was to evaluate if the Wells criteria for DVT (Table 1) [13] could be applied to the trauma population and aid clinicians in detection of DVTs.

**Table 1:** Wells criteria for the prediction of deep vein thrombosis (DVT)^a^

Clinical Characteristic	Score
Active cancer (patient either receiving treatment for cancer within the previous 6 months or currently receiving palliative treatment)	1
Paralysis, paresis, or recent cast immobilization of the lower extremities	1
Recently bedridden for ≥ 3 days, or major surgery within the previous 12 weeks requiring general or regional anesthesia	1
Localized tenderness along the distribution of the deep venous system	1
Entire leg swelling	1
Calf swelling at least 3 cm larger than that on the asymptomatic side (measured 10 cm below tibial tuberosity)	1
Pitting edema confined to the symptomatic leg	1
Collateral superficial veins (non-varicose)	1
Previously documented deep vein thrombosis	1
Alternative diagnosis at least as likely as deep vein thrombosis	-2

^a^ Wells scoring system for DVT: -2 to 0: low probability, 1 to 2 points: Moderate probability, 3 to 8 points: high probability

## Methods

This study was conducted at a Level I trauma center following review and approval by the North Memorial Institutional Review Board. All patients admitted to the trauma service at the authors’ institution from January 2012 to July 2013 who underwent venous duplex scanning (VDS) were retrospectively identified. A standardized venous thromboembolism prophylaxis protocol using sequential compression devices, and early mobilization was followed for all patients admitted to the trauma service. Additionally, routine enoxaparin (or alternative anticoagulation) was started on post admission day 1 in patients determined to be at high risk for VTE, except when contraindicated. High risk patients are defined in our protocol as having one or more of these risk factors: spinal cord injury, traumatic brain injury, complex pelvic fractures, patients requiring multiple surgeries, solid organ injury, major venous injury, immobility >72 h, previous history of VTE and cancer.

VDS was performed on iU22 xMATRIX Ultrasound System (Philips Healthcare, Andover, MA), by trained ultrasound technologists. The diagnosis of DVT was based on final VDS reporting and classified as positive or negative.

The Wells score for each patient was calculated on the day of duplex ultrasound by a blinded reviewer (critical care fellow) based on a retrospective chart review of clinical symptoms and patient history factors from physicians’, nurses’ and other medical personnel notes. Wells score was calculated using Mdcalc.com’s online calculator. Descriptive statistics and associations of the Wells score with incidence of DVT were calculated for the cohort. To calculate the sensitivity, specificity, positive predictive value (PPV) and negative predictive value (NPV) of the Wells scoring criteria, cut-off scores of 1 for low probability and cut-off scores of 2 for moderate and higher probability were used. The receiver operating characteristic (ROC) curve was analyzed and area under ROC curve (AUROCC) was used as a performance marker of the Wells scoring system in risk stratifying the trauma population.

Statistical analysis was performed using GraphPad Prism 6 (GraphPad Software Inc. La Jolla, CA). The statistical significance between proportions was determined by Chi-Square test, Mann-Whitney *U* test was used for ISS and Wells scores, correlation was calculated using Pearson *r* and coefficient of determination *R*^*2*^, and an unpaired *t-*test was used for the rest.

## Results

**Fig. 1:**
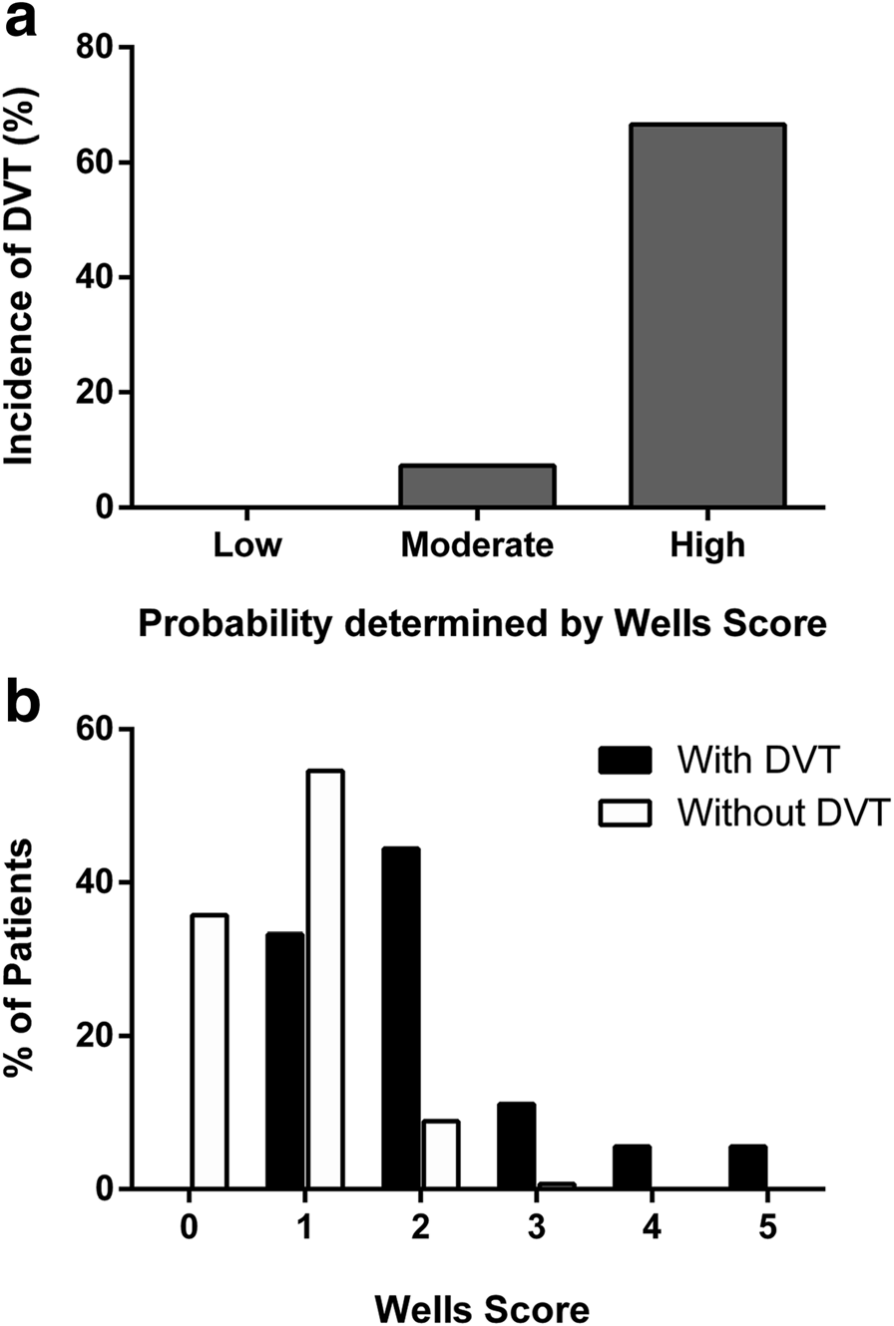
Incidence of DVT by probability estimation on the Wells scoring system: low, moderate, and high. Incidence increases with increasing risk. **a** Distribution of Wells scores in patients with and without DVT on Venous duplex surveillance (VDS). **b** Median Wells score of patients with DVT was significantly higher than the median Wells score of patients without DVT (2 vs. 1, *p* <0.0001)

A total of 2712 patients were admitted to the trauma service at the authors’ institution during the specified time period. Of these patients, the cohort consisted of 298 trauma patients, who underwent VDS for DVT evaluation. The median injury severity score of the cohort was 17 (1–66) and the median age was 58 (18–96) years. There were 197 (66 %) males and 101 (34 %) females in the cohort with a mean length of stay (LOS) of 13.5 days in the hospital and 6 days in the Intensive Care Unit (ICU) respectively. Of the 298 patients studied, a total of 18 patients (6 %) were positive for DVT. Wells score was used to define each patient’s probability of developing DVT, patients were assigned a score and then categorized: -2 to 0 points: low probability, 1 to 2 points as moderate probability, and 3 to 8 points as high probability. When stratified into the above-mentioned categories, the incidence of DVT was found to be 0 % in low probability, 7.3 % in moderate probability and 66.6 % in high probability patients (Fig. 1a). There were no significant differences in the injury severity scores (ISS), age or sex between the patients developing DVT vs. those with no DVT. Median Wells score of patients without DVT was 1(1–3) compared to a median score of 2 (1–5) in those with DVT (*p* <0.0001) (Fig. 1b). The characteristics of the cohort are provided in Table 2.

**Table 2:** Patients’ characteristics of the cohort

Parameter	All Patients	No DVT	DVT	*P* Value
Age in years (median/range)	54 (18–96)	54 (18–96)	54 (18–88)	0.99
Sex (male/female)	66 %/34 %	67 %/33 %	56 %/44 %	0.313^a^
LOS in days (mean ± SD)	13.5 (±10.5)	13.0 (±10)	21.1 (±13)	0.001
ICU stay in days (mean ± SD)	6.0 (±7.5)	5.7 (±7.1)	10.6 (±1)	0.007
ISS (median/range)	17 (1–66)	17 (1–66)	17 (5–54)	0.557^b^
Wells score (median/range)	1 (1–5)	1 (1–3)	2 (1–5)	<0.0001^b^

^a^Chi-Square test

^b^Mann-Whitney *U* test, rest compared using *t*-test; DVT: deep vein thrombosis; LOS: length of stay; SD: standard deviation; ICU: intensive care unit; ISS: injury severity score

**Fig. 2:**
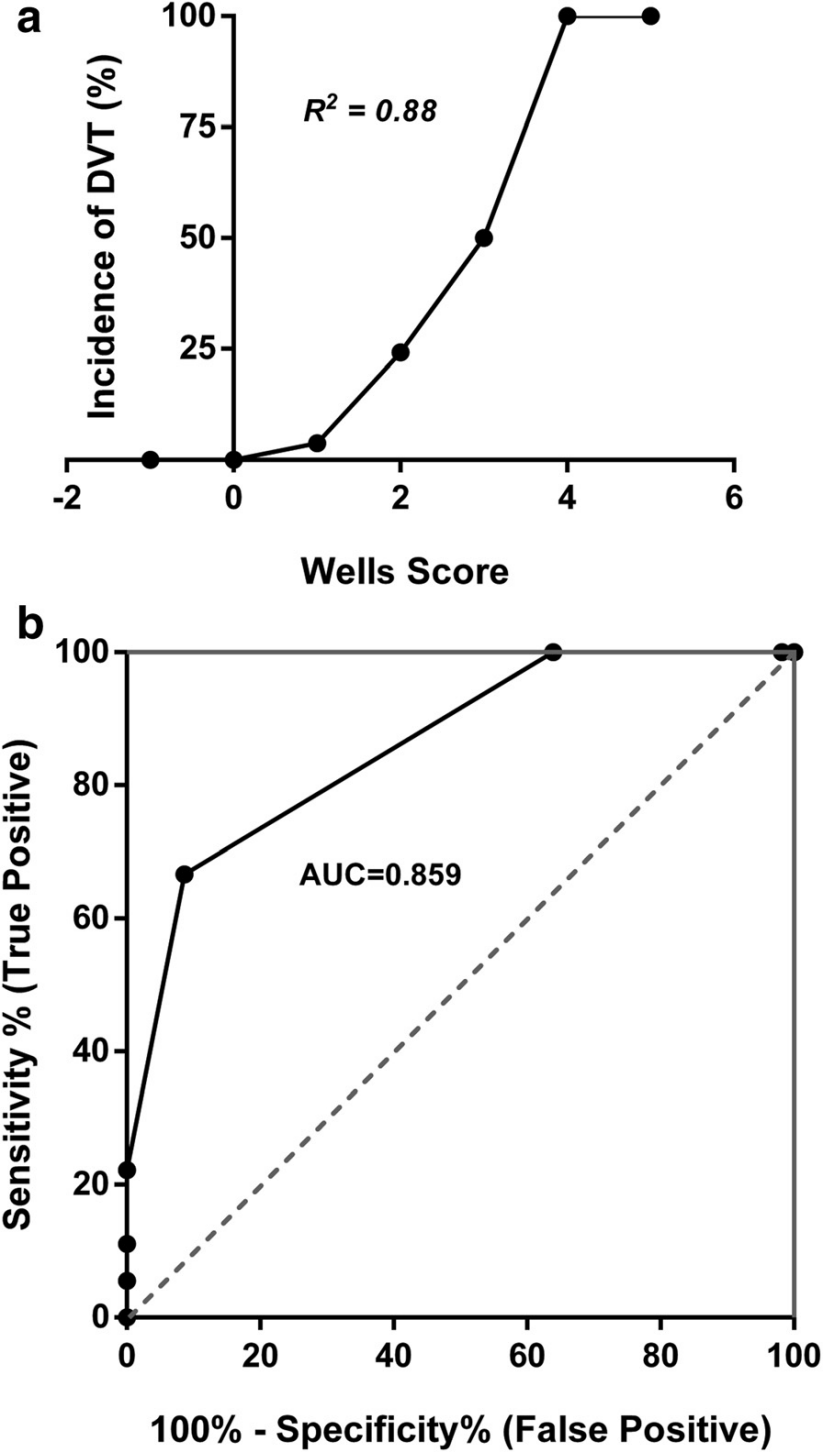
Correlation between Wells score and incidence of DVT with coefficient of determination (*R* ^*2*^ = 0.88, *p* = 0.0016) demonstrating a strong linear correlation. **a** Receiver operating characteristic (ROC) curve demonstrating the performance of Wells score in predicting likelihood of DVT. **b** Area under the ROC curve (AUROCC) value shows that high Wells scoring system is efficient in identifying the patients at risk for developing DVT based on their estimated probability after trauma

Further analysis revealed that patients with Wells score ≤ 1 had a relative risk (RR) of developing DVT of 0.075 [95 % confidence interval (CI): 0.03–0.19], while the RR of patients with Wells score of 2, 3, and 4 were 13.3 (95 % CI: 5.5–33.3), 13.9 (95 % CI: 6.5–29.8) and, 18.5 (95 % CI: 11.5–29.8) respectively. There was a strong linear correlation between Wells score and incidence of DVT with Pearson coefficient *r* = 0.94 (95 % CI: 0.64–0.99; *R*^*2*^ = 0.88; *p* = 0.0016) (Fig. 2a). In patients classified as low probability by their score (cut-off scores of 1), the Wells score was able to rule out the presence of DVT with a sensitivity of 100 % (95 % CI: 100–100 %) as well as a negative predictive value (NPV) of 100 % (95 % CI: 100–100 %) (Table 3).

**Table 3:** Statistical measures of performance of Wells score in predicting DVT in patients with cut off scores of 1

Parameter	Value	95 % CI
Sensitivity	100 %	100–100 %
Specificity	36 %	30–41 %
PPV	9 %	5–13 %
NPV	100 %	100–100 %

*DVT* deep vein thrombosis, *CI* confidence interval, *PPV* positive predictive value, *NPV* negative predictive value

In patients classified as moderate or higher probability for DVT (cut-off scores of 2), the Wells score was able to detect patients at risk of developing DVT with a specificity of 90 % (95 % CI: 87–94 %), sensitivity of 67 % (95 % CI: 45–88 %), positive predictive value of 31 % (95 % CI: 16–45 %) and NPV of 98 % (95 % CI: 96–99 %) (Table 4).

**Table 4:** Statistical measures of performance of Wells score in predicting DVT in patients with cut off scores of 2

Parameter	Value	95 % CI
Sensitivity	67 %	45–88 %
Specificity	90 %	87–94 %
PPV	31 %	16–45 %
NPV	98 %	96–99 %

*DVT* deep vein thrombosis, *CI* confidence interval, *PPV* positive predictive value, *NPV* negative predictive value

Efficacy of risk stratification based on Wells score was analyzed by receiver operating characteristic (ROC) curve analysis, which demonstrated that this scoring and stratification system accurately identifies patients with a greater likelihood of developing DVT after sustaining acute trauma. The area under ROC curve (AUROCC) was 0.859 (95 % CI: 0.77–0.95; *p* <0.0001) (Fig. 2b).

## Discussion

This study examined the utility of the Wells score for predicting DVT in patients who were admitted to the trauma service from January 2012 to July 2013 and underwent VDS. Wells score has been in use for more than a decade and has a predictive value in determining DVT risk in hospitalized patients [1, 9, 13, 14], but its efficacy specifically in trauma patients has not been studied. We have shown that patients after sustaining acute injuries can be categorized as low probability and moderate-high probability for developing DVT using the Wells scoring system and the incidence of DVT increases with the increasing score. The Wells score linearly correlated with the incidence of DVT in these patients.

Routine surveillance VDS is widely used in many trauma centers for the diagnosis of DVT, however, there is a lack of standardized DVT screening system introducing a surveillance bias [8, 15] in reporting the incidence of DVT. There is also some controversy concerning the appropriate use of VDS with some studies citing the high cost of VDS with relatively low yield of clinical findings [7]. In our study, we found that a Wells score of <1 effectively ruled out the possibility of DVT with a sensitivity and NPV of 100 %, which may eliminate the need of routine VDS in fairly large cohort of patients (33.5 %). Although the scoring was very sensitive in predicting the development of DVT, its specificity was decreased in moderate and high probability patients. This may be due to the use of strict and aggressive thromboprophylaxis protocol by trauma services, which leads to a decrease in overall rate of DVT. The retrospective design of the study precluded the ‘nonintervention’ control arm, but the ROC curve analysis with AUROCC of 0.859 (traditional academic point system rating: good) illustrated that Wells score is able to accurately stratify the trauma patients in different DVT risk categories. A Wells score of <1 very efficiently rules out the possibility of DVT with a NPV of 100 %, thus making it an effective pretest scoring system, while a Wells score of >2 rules in a possibility of DVT with a specificity of 90 %. This risk scoring system can allow judicious use of VDS and enhanced patient-directed care with reduced costs and morbidity.

There are other VTE risk assessment models that have been created specifically for trauma patients, namely the Trauma Embolic Scoring System (TESS) [16] and the Risk Assessment Profile (RAP) [17]. Although these models have been shown to be useful risk assessment tools [16, 18], they fail to accurately stratify a significant number of patients [19, 20]. This failure probably stems from few inherent limitations of these models. The cut off threshold for defining “low risk” in these models allows at least two (or more) variables to be present in trauma patients whereas presence of even one variable (Score ≥1) in the Wells scoring system qualifies a patient for further DVT surveillance. Secondly, TESS does not take into account some of the variables associated with a hypercoagulable state, while RAP uses complex array of parameters, some of which may not be feasible to measure accurately in an acute trauma setting [20]. We believe these limitations are circumvented up to an extent in Wells scoring system, which is both easy to use and takes into account risk factors predisposing to a hypercoagulable state.

There are some limitations to the current study. The Wells score accuracy may have been affected if the patients’ history and symptoms were not recorded accurately. As with the validation of all other scoring systems, this study of validation of Wells score is retrospective and a prospective analysis to further validate our findings will help establish the efficacy of this scoring system.

## Conclusion

**Fig. 3:**
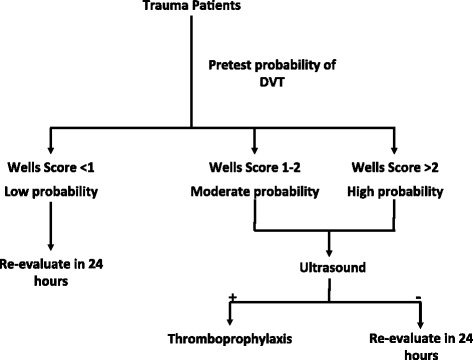
Flowchart of the protocol demonstrating potential use of Wells criteria for DVT surveillance and risk stratification in trauma patients

A Wells score of <1 in the trauma population can reliably rule out DVT and thus avoid further workup. As the Wells score increases, the risk of DVT increases linearly with it, establishing it as a valid pretest tool for risk stratification. Patients sustaining traumatic injuries can be stratified using Wells score into low, moderate and high probability of DVT, as shown in Fig. 3.

## Abbreviations

AUROCC, area under receiver operating curve; CI, confidence interval; DVT, deep vein thrombosis; ICU, intensive care unit; ISS, injury severity score; LOS, length of stay; NPV, negative predictive value; NTDB, National Trauma Data Base; PE, pulmonary embolism; PPV, positive predictive value; RAP, risk assessment profile; ROC, receiver operating characteristic; TESS, Trauma Embolic Scoring System; VDS, venous duplex scanning; VTE, venous thromboembolism

## Additional file

Additional file 1: Wells Scoring for patients. (XLSX 25 kb)
